# Poplar stem transcriptome is massively remodelled in response to single or repeated mechanical stimuli

**DOI:** 10.1186/s12864-017-3670-1

**Published:** 2017-04-17

**Authors:** Lise Pomiès, Mélanie Decourteix, Jérôme Franchel, Bruno Moulia, Nathalie Leblanc-Fournier

**Affiliations:** Université Clermont Auvergne, INRA, PIAF, F-63000 Clermont-Ferrand, France

**Keywords:** Mechanotransduction, Time series, Acclimation, Accommodation, microarray, Thigmomorphogenesis, Abiotic stress, Mechanical stimuli

## Abstract

**Background:**

Trees experience mechanical stimuli -like wind- that trigger thigmomorphogenetic syndrome, leading to modifications of plant growth and wood quality. This syndrome affects tree productivity but is also believed to improve tree acclimation to chronic wind. Wind is particularly challenging for trees, because of their stature and perenniality. Climate change forecasts are predicting that the occurrence of high wind will worsen, making it increasingly vital to understand the mechanisms regulating thigmomorphogenesis, especially in perennial plants. By extension, this also implies factoring in the recurring nature of wind episodes. However, data on the molecular processes underpinning mechanoperception and transduction of mechanical signals, and their dynamics, are still dramatically lacking in trees.

**Results:**

Here we performed a genome-wide and time-series analysis of poplar transcriptional responsiveness to transitory and recurring controlled stem bending, mimicking wind. The study revealed that 6% of the poplar genome is differentially expressed after a single transient bending. The combination of clustering, Gene Ontology categorization and time-series expression approaches revealed the diversity of gene expression patterns and biological processes affected by stem bending. Short-term transcriptomic responses entailed a rapid stimulation of plant defence and abiotic stress signalling pathways, including ethylene and jasmonic acid signalling but also photosynthesis process regulation. Late transcriptomic responses affected genes involved in cell wall organization and/or wood development. An analysis of the molecular impact of recurring bending found that the vast majority (96%) of the genes differentially expressed after a first bending presented reduced or even net-zero amplitude regulation after the second exposure to bending.

**Conclusion:**

This study constitutes the first dynamic characterization of the molecular processes affected by single or repeated stem bending in poplar. Moreover, the global attenuation of the transcriptional responses, observed from as early as after a second bending, indicates the existence of a mechanism governing a fine tuning of plant responsiveness. This points toward several mechanistic pathways that can now be targeted to elucidate the complex dynamics of wind acclimation.

**Electronic supplementary material:**

The online version of this article (doi:10.1186/s12864-017-3670-1) contains supplementary material, which is available to authorized users.

## Background

In their fluctuating environment, plants are constantly exposed to abiotic stimuli to which they are sensitive and responsive. Some of these stimuli, such as wind exposure, count a strong mechanical component that has a major influence on plant growth and development. Exposure to such mechanical stimulations results in the so-called thigmomorphogenetic syndrome characterized by a reduction in stem elongation [1, 2], local stimulation of radial growth [3, 4], and modification of the stem’s mechanical properties [5]. These alterations of plant architecture are thought to improve plant acclimation to chronic wind regimes [6]. Using continuous monitoring techniques [4, 7], the early kinetics of these plant growth responses were studied in tomato and poplar by applying quantified stem bending. In poplar, a single transitory stem bending led first to a short period of secondary growth inhibition (4 h) followed by a massive stimulation of the growth rate over 3 days, and finally a relaxation to normal values [4].

To better understand tree acclimation to wind, it is important to first unravel the processes that regulate thigmomorphogenetic syndrome. However, the way plant cells perceive and transduce mechanical signals is still poorly understood. Two major classes of potential mechanosensors are thought to be involved: plant MechanoSensitive (MS) ion channels and Receptor-Like Kinases (RLK) inserted into the cell wall–plasma membrane–cytoskeleton continuum (see [8] for review). Prior to the advent of transcriptomics, studies had identified a handful of mechanoresponsive genes, including *TOUCH* genes (*TCH*) that mainly encode calmodulins or calmodulin-like proteins and Xyloglucan endo-Transglycosylase/Hydrolase (XTH) [9], genes encoding protein kinases [10, 11], Transcription Factors (TF) [12], genes involved in Jasmonic Acid (JA) and ethylene synthesis [13, 14], and genes involved in antioxidative responses [15]. In 2005, a transcriptome analysis of touch-stimulated *Arabidopsis* rosette leaves allowed a more global insight into the molecular functions altered after a touch-stimulus. Over 700 genes presented regulated expression 30 min after the stimulus [16], representing over 2.5% of the genome. Among these genes, the vast majority (589/760) were up-regulated. Analysis of the functional categorization of these up-regulated genes revealed enrichment in genes encoding calcium-binding proteins, cell-wall proteins, disease resistance proteins, kinases and TF, and a decline in genes involved in general metabolism and the ubiquitin/protein degradation pathway. For the down-regulated genes, the “transcription factor” and “cell-wall-associated protein” categories were over-represented. In a subsequent transcriptomic study of the effect of a long-term exposure (8 weeks) to low-speed wind in *Populus nigra* leaves, Fluch et al. found at least 98 up- and 94 down-regulated genes [17], including genes encoding cell-wall modification proteins, proteins with regulatory roles (e.g. kinases, calmodulin, *etc.*), Reactive Oxygen Species (ROS) producing or scavenging proteins, or constituents of microtubules. Unfortunately, these two transcriptomic studies concern a unique time-point after stimulation, giving a very static view of the molecular response to mechanical stimulus. In 2004, Kimbrough *et al*. found 1,691 mechanoresponsive genes on root apices that were transiently (5 s time-lapse) stimulated by moving them back and forth and harvested at different times (0, 2, 5, 15, 30, and 60 min post-stimulation) [18]. To our knowledge, this is the only in-depth kinetic (time-series) study ever made in plants on responses to mechanical stimuli. However, these data cannot be viably extrapolated to the responses of aboveground organs, as the soil and aerial mechanical environments, and the selective pressures they apply to the corresponding organs, are highly distinct.

Despite the tentative general flowchart of physiological and molecular responses to mechanoperception established by Telewski [19], we still have no general understanding of molecular mechanoresponse. The fact is that most molecular thigmomorphogenesis studies were realized on different plant species, submitted to different types of mechanical stimulations, with a huge majority of them using uncontrolled mechanical loadings, all of which makes cross-comparison difficult.

There is an even more striking lack of molecular information for recurring mechanical loads such as wind episodes [6, 20]. In 2010, Martin et al. assessed poplar responses to multiple successive stem bendings mimicking wind, and provided the first evidence of a process of desensitization to mechanical loads at the growth and molecular levels [21]. They found that four early mechanoresponsive genes —encoding two calmodulins, a C2H2 TF, *i.e. PtaZFP2*, and a XTH, respectively— responded with lower intensity to a second stem bending, applied 24 h after a first one. This phenomenon is now referred to as ‘accommodation’ and is thought to be crucial to avoid over-response to wind [6, 22]. In trees, transcriptomic analysis of transgenic poplars overexpressing *PtaZFP2* constituted a first step toward the characterization of this phenomenon at molecular level. This study suggested that part of the accommodation process could be explained by a regulation at transcriptional level [6], thus requiring a more global analysis.

Deciphering the molecular mechanisms driving poplar accommodation to bending stimulation could be crucial to help better understand how trees acclimate to wind. In this study, our aim was to identify new molecular actors involved in mechanoresponse and the accommodation process, and unravel their kinetics of contribution. We thus conducted a time-series search of the transcriptome changes in poplar stem tissue subjected to one or two successive bendings.

## Results

### Kinetic analysis of the transcriptomic responses to a single bending in poplar stem

To unravel the complexity and dynamics of the transcriptomic response of poplar stem to a bending stimulus, we performed microarray gene expression profiling at 0.5 h, 2 h, 24 h or 72 h after a tightly controlled bending.

**Fig. 1:**
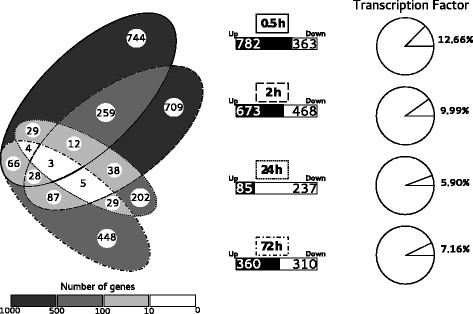
Global analysis of DEG after bending in poplar stem. Venn diagram shows the distribution and the overlaps of DEG at 0.5, 2, 24 and 72 h PB. 2,633 different DEG were identified. Numbers in the central part indicate the number of repressed and induced genes at each time-point. Proportion of DEG corresponding to TF oscillates between 13 and 6%

Statistical analysis revealed 2,663 genes differentially expressed along the time-course (Fig. 1; Additional file 1). These Differentially Expressed Genes (DEG) represented 6% of the whole poplar genome, and 75.6% of them were regulated at just 0.5 h or 2 h post-bending (0.5 h PB or 2 h PB). A third of the DEG observed at 0.5 h PB keep being differentially expressed at 2 h PB. At these earlier time-points, a higher number of DEG was up-regulated. At 24 h PB, the transcriptomic response concerned a smaller set of genes (312 DEG), most of which were down-regulated. At 72 h PB, the number of DEG was equally distributed between up- and down-regulated categories.

As a first examination of the mechanical regulatory network, we considered for each time-point the transcripts presenting the higher amplitude of regulation (Additional file 2). At 0.5 h PB, the 10 most highly up-regulated genes encompassed genes already known to present a short-term response to mechanical stimuli, such as an ortholog of a XTH (Potri.018G095200) [17], or *PtaZFP2* [23]*,* a poplar C2H2 TF (Potri.001G235800). Strongly affected expression was also found for two other TF: one that was related to ethylene signalling (Potri.004G220400) and the other that was an ortholog of RRTF (Potri.009G101900), a redox-responsive member of the Ethylene Response Factor (ERF) TF subfamily [24]. Interestingly, highly up-regulated genes also included homologs of the Late Embryogenesis Abundant (LEA) hydroxyproline-rich glycoprotein family (e.g. Potri.006G204300, Potri.009G158900), including *NHL10/NDR1,* a plasma membrane-embedded protein involved in mechanisms maintaining plasma membrane-cell-wall adhesions in *Arabidopsis* [25].

At 2 h and 72 h PB, the most highly up-regulated genes include many genes related to cell-wall modifications or wood formation. For example, three of the 10 most highly up-regulated genes at 2 h PB (Potri.004G210600, Potri.012G015000, Potri.009G012100) encoded homologs of *Arabidopsis* fasciclin-like arabinogalactan proteins (FLA) putatively involved in stem biomechanics in plants [26]. At 72 h PB, Potri.009G012200 and Potri.011G161500, encode, respectively; a FLA protein and a berberine bridge enzyme shown to play a role in lignin monolignol metabolism in *Arabidopsis* [27].

At 24 h PB, the set of genes most strongly up- or down-regulated are predicted to encode proteins involved in cellular metabolism, such as a Homocysteine S-methyltransferase (Potri.008G155900), antioxidant components (Potri.003G066400, Potri.006G141400, Potri.011G140400) or elements of ribosome biogenesis (Potri.014G135600 and Potri.015G079600).

A gene highly down-regulated (Potri.010G069000) at 0.5 h, 2 h, 72 h PB (and to a lesser extent 24 h PB) matched to a homolog of *Arabidopsis PHOSPHATE1* (*PHO1*) encoding a Pi exporter [28], pointing, for the first time, to a role of Pi homeostasis in mechanoresponse.

### Transcriptional regulation of mechanoresponsive TF-encoding genes

**Fig. 2:**
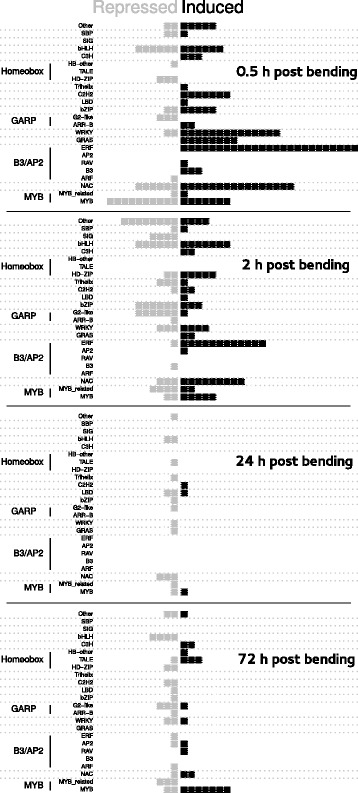
List of transcription factor families whose members are differentially expressed in response to bending. The PlantTFDB database [29] was used to identify the TF-encoding genes and their family among DEG. The family assignment rules based on the detection of pre-set domains in gene sequences identified 33 TF families (among the 58 different families defined for poplar in PlantTFDB). When a TF family appeared less than 5 times in DEG, it was classified into the “other” category. The SIG family, not present in PlantTFDB, was revealed by Phytozome data

Among the 2,663 mechanoresponsive genes, 271 corresponded to known or putative TF. As shown in Fig. 1, the proportion of TF varied over the time-course, representing 12.66% of the DEG at 0.5 h PB but only 5.90% at 24 h PB. These mechanoregulated TF were classified into 34 of the 58 TF families described in the *PlantTF* database [29]. The TF families were differentially represented depending on time post-bending (see Fig. 2). At 0.5 h PB, NAC-type TF represented a significant fraction of the regulated TF (22 members), whereas only 5 NAC-type TF were differentially regulated at 72 h PB. Other TF families involved in stress responses were highly represented at early time-points, as illustrated by the 16 WRKY-type TF and the 38 members of the ERF family. Conversely, TF members of the TALE family were involved at later time-points (24 h and 72 h PB). Members of the MYB family were also highly represented among the mechanoresponsive TF, being present both at early and late time-points, and either up- or down-regulated. Three members of this MYB family show an interesting pattern of expression along the time-course by being down-regulated at 0.5 h and up-regulated at 72 h. These poplar genes (Potri.003G13200, Potri.007G106100 and Potri.009G053900) respectively encode orthologs of MYB103, MYB69 and MYB46 *Arabidopsis* TF, known to be involved in the regulation of secondary cell wall biogenesis [30].

### Expression clustering and Gene Ontology analysis of mechanoresponsive genes in poplar

**Fig. 3:**
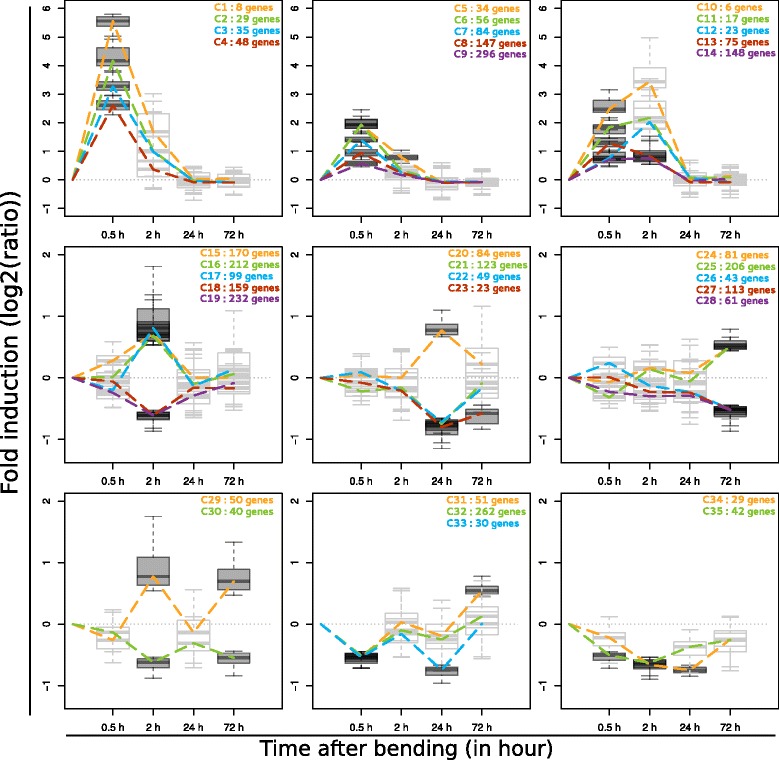
K-means clustering of gene expression data. A k-means clustering was performed on DEG from the time-course and two-bendings experiments. For each cluster, at each time -point, a boxplot represent the dispersion of the log2-converted mean fold-change (bent *vs.* unbent poplars) for all the genes of the cluster. For graphical clarity, the different clusters were manually re-grouped according to their mean expression profile

To gain insight into the biological processes underlying poplar response to bending, two approaches were combined: gene expression clustering and Gene Ontology (GO) enrichment. Using the k-means partitioning method, the expression profiles of the 2,663 DEG were distributed into 35 clusters, each represented in Fig. 3 by their average gene expression. This clustering approach revealed a high diversity of expression profiles, many of them supported by a high number of genes (apart from clusters 1 and 10). The most striking expression pattern, shown by 11 of the clusters, was an early and transitory induction at 0.5 h PB. The most highly up-regulated genes are included in these same clusters (Fig. 3). A smaller number of genes showed an initial down-regulation of expression followed by reversion to basal levels (e.g. cluster 35). Clusters 5, 13, 23, 34, and 35 regrouped genes that were differentially expressed at more than two time-points across the time-series. Some interesting expression patterns are also shown by cluster 31 regrouping genes that were down-regulated at 0.5 h PB then up-regulated at 72 h PB, and clusters 29, 30 and 33 regrouping genes showing a regulation throughout the time-course. To find out whether these co-expressed genes could be indicative of putative biological function, each cluster was analysed for enrichment of GO terms in the Biological Process (BP), Molecular Function (MF) and Cellular Component (CC) categories (Additional file 3). A clear picture delivered by this GO analysis is the early activation of responses linked to “defence” or “abiotic stress” shown by different GO enriched-terms found for clusters 1, 2, 3, 4, 6, 7, 8, 12 and 14. Three of the four clusters regrouping the earlier, transitory and most up-regulated genes showed an over-representation of terms related to transcription regulation or TF in both the BP and MF categories (clusters 2, 3, and 4). In contrast, cluster 10 is annotated with GO terms related to “negative regulation of transcription”. This GO analysis also argues in favour of an early triggering of a “signal transduction” (cluster 5 and 12) involving protein phosphorylation (cluster 8 and 9) and positive regulation of the MAPK cascade (cluster 5). The early-up-regulated genes counted a high prevalence of GO terms related to plant hormones, especially ethylene (see GO:0009873 “ethylene-activated signalling pathway” for clusters 4 and 5) and JA (for example, see GO:0009753 “response to jasmonic acid” for clusters 11 and 13). Interestingly, clusters composed of genes down-regulated 2 h PB (clusters 18 and 19) were enriched with genes related to photosynthesis or plastid function. For clusters of late-up-regulated genes (72 h PB), the GO BP analysis suggests the involvement of genes contributing to remodelling of the cell wall. For example, clusters 24, 25 and 31 are annotated with the “plant-type cell-wall organization or biogenesis” (clusters 24 and 25, GO:0071669) or “regulation of secondary cell-wall biogenesis” (cluster 31, GO: 2000652) terms. Given its enrichment with terms related to “microtubule-binding” (GO:0008017) and the importance of microtubules in the build-up of the cell wall [31, 32], cluster 29 could also contain genes that are central to this cell-wall remodelling process.

### Time-course expression analysis of candidate genes by qRT-PCR

**Fig. 4:**
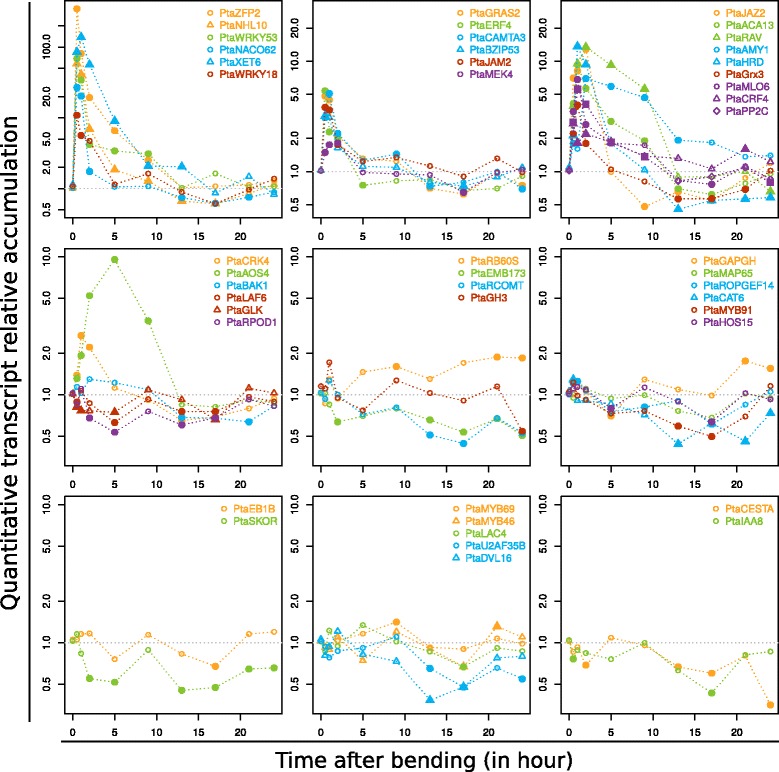
Expression profiles of selected genes along a refined time-course. Total RNAs were extracted from the stems of control plants and from bent stems at different times (0.5, 1, 2, 5, 9, 13, 17, 21 and 24 h) after bending. Changes in the abundance of the transcripts were analysed by qRT-PCR using specific primers (Additional file 4). Fold-changes are quantified in comparison to control plants collected at identical time-points. Plain symbols correspond to a significantly different transcript accumulation in bent plants according to a Mann–Whitney-Wilcoxon test

A RT-qPCRs experiment was performed with the dual aim of validating the reliability of our microarray experiment and refining the dynamics of the biological processes triggered in poplar after stem bending. Expression of 46 selected genes was analysed along a new time-series made with samples collected at regular intervals between 0.5 and 24 h PB (Fig. 4). Each gene was selected to be representative of a cluster on the basis of its expression profile and its putative biological function as determined by GO categorization (Additional file 3).

Globally, except for one gene (*PtaEB1B*), the PCR data backs up the microarray analysis. Moreover, the refined time-series revealed an even greater complexity of the responses. Given the predominance of GO terms related to “defence response” in our data, we studied a variety of genes involved in this biological process, typically members of the WRKY, NAC, GRAS, C2H2 and ERF TF families. For some of these, the up-regulation observed at early time-points (0.5 h and 2 h PB) is maintained until 9 h PB (e.g. *PtaZFP2*, *PtaNHL10*, *PtaXET6*, *PtaWRKY53*, *PtaRAV*) whereas others show a more transient induction confined to the very first hours after bending (e.g. *PtaWRKY18, PtaNACO62, PtaGRAS, PtaBZIP53, PtaERF4*). Results considering several molecular components of calcium/ROS/Phosphorylation also showed contrasted patterns. Whereas the tested genes involved in phosphorylation cascade signalling (*PtaMEK4, PtaCRK4, PtaPP2C*) or calcium signalling (*PtaCAMTA3*) were transiently induced at early stages (before 5 h PB), the up-regulation of *PtaACA13,* encoding a putative calcium-transporting ATPase enzyme involved in the export of Ca^2+^, was maintained until 9 h PB. An unexpected result obtained with this PCR analysis was the down-regulation of a high number of early-induced genes at late stages (after 13 h) (e.g. *PtaHRD, PtaACA13* or *PtaGXR3*). Considering hormonal signalling, our results suggested that *PtaIAA8*, homologous to an auxin-regulated TF, and *PtaCESTA*, homologous to bHLH TF involved in Brassinosteroid biosynthesis, were down-regulated in a biphasic manner. For JA signalling, *PtaJAM2*- and *PtaJAZ2*-like, two predicted JA-induced TF, showed a high but very transient induction within the first 2 h PB. In contrast, the peak accumulation of transcripts of *PtaAOS4*, a gene encoding an allene oxide synthase probably involved in the JA biosynthetic pathway, was shifted to later stages (5 h–9 h PB). Another set of genes (e.g. *PtaLAF6*, *PtaRPOD1*, *PtaGLK, PtaSKOR*) was rapidly down-regulated (1 h PB) and still repressed until 17 h PB. These genes encompassed functions related to cellular and metabolic processes, such as regulation of photosynthetic processes (*PtaLAF6*, *PtaRPOD1* and *PtaGLK*) or cellular homeostasis, as illustrated by *PtaSKOR*, an ortholog of an *Arabidopsis* gene encoding a Shaker family K^+^ channel. Finally, the genes related to secondary cell-wall biogenesis (*PtaMYB69*; *PtaMYB46*), lignin synthesis (*PtaRCOMT1*; *PtaLAC4*) or microtubule dynamics (*PtaMAP65*) were repressed until around the 17th hour before recovering to basal levels.

**Fig. 5:**
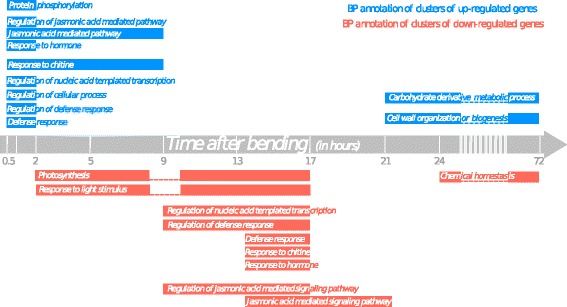
Time-course of the biological processes involved in response to bending. These annotations correspond to the GO terms that are enriched in clusters of gene expression. Biological Processes are time-placed to match the expression of representative genes

Combined with GO analysis of the clusters, this time-course analysis provides a new picture of the molecular responses occurring in poplar stems in response to mechanical stimulation that was drawn in a simplified model of over-represented GO terms (BP and MF) in Fig. 5.

### Genome-wide evaluation of the effect of successive mechanical stimulations

**Fig. 6:**
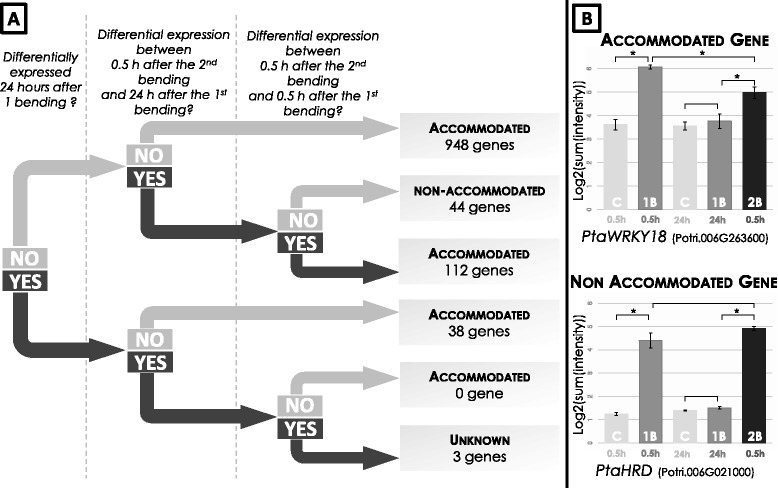
Decision tree used to classify the genes into the “accommodated” or “non-accommodated” gene categories. **a** For each gene differentially regulated at 0.5 h PB, “YES” or “NO” status was determined by the statistical test resulting from the microarray analysis described above. **b** Gene examples for the “accommodated” or “non-accommodated” gene categories. The *PtaWRKY18* and *PtaHRD* genes are differentially expressed at 0.5 h PB but not at 24 h PB, whereas the expression of *PtaHRD* is similarly regulated by the two successive bending while*, PtaWRKY18* expression is less affected by the second bending than the first one. According to the decision tree, *PtaWRKY18* is classified into the “accommodated gene” category, and *PtaHRD* into the “non-accommodated gene” category

To unravel the genome-wide impact of successive mechanical stimuli, we analysed the effect of a second bending performed 24 h after a first one. As shown in Fig. 6a, the genes presenting a modified expression due to the second bending were revealed through a statistical test by comparing their expression levels 0.5 h after the second bending with their expression at 24 h after the first bending. We then compared the level of regulation of these DEG against to their regulation observed at 0.5 h after a single bending. The different gene profiles were then classified through a decision-tree approach (Fig. 6a).

Several regulation patterns were observed. The results showed that among the 1,104 genes transiently regulated at 0.5 h after a single mechanical stimulation, 986 were unaffected by the second bending and 112 were less regulated (e.g. *PtaWRKY18* (Potri.006G263600) Fig. 6b, “accommodated gene”). Among the 41 early-regulated genes that were still differentially expressed at 24 h after the first bending, 38 were not regulated by the second treatment and, for 3 genes, the analysis was unable to classify them. Overall, this means that 96% of the DEG regulated after the first mechanical stimulation are not any more or less affected by the second one.

This analysis also revealed 19 newly-regulated genes – *i.e.* genes that had shown no impact after a single mechanical treatment but were differentially regulated 0.5 h after the second bending (Additional file 5). Finally, among the 1,145 genes regulated by a single treatment, only 44 genes were similarly regulated after the second bending (e.g. *PtaHRD* (Potri.006G021000) in Fig. 6b). As shown in Table 1, these “non-accommodated genes” were mainly distributed into three of the expression clusters previously defined in the time-series analysis: *i.e.* clusters 9, 10 and 14, grouping 9, 12, and 10 of these genes respectively. These co-expression profiles could suggest a common regulation.

**Table 1:** List of the “non-accommodated” genes after two successive bendings of young poplar stem

Cluster	Id_Gene Poplar	Log2 expression fold-change		Id_Gene Ath	Ath Description (Alias)
		1B0.5/C	2B0.5/1B24		
4	Potri.010G041300	2.92	1.22	*NULL*	*NULL*
6	Potri.T131200	2.21	1.64	AT3G19680	Protein of unknown function (DUF1005)
6	Potri.008G159300	2.03	1.85	AT3G22930	Calmodulin-like 11 (CML11)
7	Potri.004G086900	1.28	0.78	*NULL*	*NULL*
7	Potri.006G055500	1.39	1.24	*NULL*	*NULL*
7	Potri.009G084000	1.38	1.24	AT1G10020	Protein of unknown function (DUF1005)
7	Potri.009G092900	1.44	1.2	AT2G14960	Auxin-responsive GH3 family protein (GH3.1)
7	Potri.016G132500	1.42	0.93	AT3G51710	D-mannose binding lectin protein with Apple-like carbohydrate-binding domain
8	Potri.001G309600	0.89	1.07	AT1G49230	RING/U-box superfamily protein
8	Potri.006G110000	1.2	0.78	AT3G51550	Malectin/receptor-like protein kinase family protein (FER)
8	Potri.006G141500	0.95	0.91	AT2G05940	Protein kinase superfamily protein
8	Potri.008G126800	1.12	0.89	AT2G01100	unknown
8	Potri.009G030600	0.97	0.81	AT2G28890	Poltergeist like 4 (PPL4)
8	Potri.010G081100	1.31	1.07	AT3G22910	ATPase E1-E2 type family protein / haloacid dehalogenase-like hydrolase family protein
8	Potri.012G126100	1.17	0.88	AT5G51460	Haloacid dehalogenase-like hydrolase (HAD) superfamily protein (TPPA)
8	Potri.018G093600	1.22	0.94	AT5G57480	P-loop containing nucleoside triphosphate hydrolases superfamily protein
8	Potri.019G128100	0.93	1.01	AT2G30360	SOS3-interacting protein 4 (CIPK11)
9	Potri.001G344100	0.52	0.82	AT5G14500	Aldose 1-epimerase family protein
9	Potri.005G158400	0.59	0.79	AT1G02070	unknown
9	Potri.006G235800	0.73	0.98	AT5G25930	Protein kinase family protein with leucine-rich repeat domain
9	Potri.008G103300	0.64	0.83	AT5G49520	WRKY DNA-binding protein 48 (WRKY48)
9	Potri.010G132800	0.64	0.85	AT3G25600	Calcium-binding EF-hand family protein
9	Potri.013G115700	0.51	0.79	AT1G34300	Lectin protein kinase family protein
9	Potri.015G063500	0.76	0.78	AT3G47730	ATP-binding cassette A2 (ABCA2,ATH1)
9	Potri.017G000700	0.81	0.89	AT2G44130	Galactose oxidase/kelch repeat superfamily protein
10	Potri.006G021000	3.15	3.4	AT2G36450	Integrase-type DNA-binding superfamily protein (HRD)
11	Potri.012G048200	1.28	1.07	AT1G18210	Calcium-binding EF-hand family protein
12	Potri.002G126300	0.7	1.09	AT4G25000	Alpha-amylase-like (AMY1)
12	Potri.004G112700	0.83	1.48	AT5G15720	GDSL-motif lipase 7 (GLIP7)
13	Potri.005G056800	1.24	0.85	AT5G60900	Receptor-like protein kinase 1 (RLK1)
13	Potri.018G076500	1.31	0.84	AT3G24440	Fibronectin type III domain-containing protein (VIL1,VRN5)
14	Potri.003G159800	0.99	1.2	AT5G47910	Respiratory burst oxidase homologue D (RBOHD)
14	Potri.005G015300	0.9	0.86	*NULL*	*NULL*
14	Potri.005G055200	0.88	1.06	AT5G28680	Malectin/receptor-like protein kinase family protein (ANX2)
14	Potri.006G187900	0.89	0.82	AT2G24300	Calmodulin-binding protein
14	Potri.006G219100	1.03	1.04	AT1G72510	Protein of unknown function (DUF1677)
14	Potri.009G002200	0.82	0.95	AT5G22860	Serine carboxypeptidase S28 family protein
14	Potri.010G240800	0.5	0.89	AT5G04760	Duplicated homeodomain-like superfamily protein
14	Potri.013G125000	0.51	1.04	AT3G54420	Homolog of carrot EP3-3 chitinase (CHITIV)
14	Potri.013G130000	1.02	0.97	AT1G53050	Protein kinase superfamily protein
14	Potri.014G111800	0.67	1.23	AT4G01700	Chitinase family protein
14	Potri.017G013300	0.87	1.09	AT4G28400	Protein phosphatase 2C family protein
15	Potri.006G101400	1.75	1.5	AT5G01380	Homeodomain-like superfamily protein

These genes are differentially expressed 0.5 h after a single bending and their regulation is not different whether one or two successive bendings are applied. Id_gene corresponds to the gene model found in the Phytozome database [55] for poplar or Arabidopsis (Ath). Log2 expression fold-change is shown for the different analyses: expression fold-change 0.5 h after a single bending compared to a control plant (1B0.5 h/Control) and expression fold-change after a second bending compared to the 24 h post-bending time-point (2B0.5 h/1B24h)

Interestingly, among the 44 “non-accommodated” genes, several are involved in maintaining the functional integrity of the plant cell wall after biotic/abiotic stresses in other species. Indeed, several genes are predicted to encode members of the CrRLK1 family of RLK in *Arabidopsis*, such as Potri.006G110000, an ortholog of FERONIA recently described as a putative mechanosensor in *Arabidopsis* [33], but also ANX2 (Potri.005G055200) and RLK1 (Potri.005G056800). Furthermore, among the other “non-accommodated” genes, some were described as components of signalling cascades coordinating cellular responses to help maintain plant cell-wall integrity [34]. This is the case for Potri.003G159800 encoding the respiratory burst oxidase homolog D, responsible in *Arabidopsis* for apoplast ROS production [35] or for genes encoding calcium-related proteins such as calcium-binding EF-hand family protein (Potri.012G048200, Potri.010G132800), Calmodulin-binding protein (Potri.006G187900 Potri.008G159300) or CBL-interacting protein kinase (Potri.019G128100).

## Discussion

### Short- and long-term impacts of transient stem bending on transcriptional responses in poplar

Lee et al. studying the transcriptomic effect of a touch stimulus at a single time-point (30 min) in the herbaceous *Arabidopsis* showed that the expression of over 2.5% of the *Arabidopsis* genome was up-regulated at least twofold [16]. Here, in poplar, a single transitory bending modulated the expression of about 6% of the genome over 4 time-points, and strikingly, the majority of changes in gene expression were observed within the first two hours post-bending. Such massive early response has been seen in other transcriptomic time-course studies of responses to stress (e.g. defence response to *Botrytis cinerea* in [36]). Moreover, at 0.5 h PB, genes are mainly up-regulated (782 up- *vs.* 362 down-regulated genes). A high proportion of these early-regulated genes showed no differential regulation at 2 h PB and thereafter. The transcripts they produce could be classified as unstable transcripts according to Gutierrez *et al.* [37]. Actually, about 20% of the unstable transcripts described by these authors were up-regulated at 0.5 h PB in our experiment (data not shown).

Our study illustrates the massive extent of transcriptomic changes in response to a non-wounding mechanical stimulus, but also sheds new light on the duration and dynamics of the phenomenon. The refinement of the time-course by qPCR for 46 candidate genes brought key insight by revealing unexpected short or long-lasting expression profiles. Notably, this analysis highlighted a high number of early-induced genes presenting inhibited expression at later time-points. This suggests the existence of an active negative feedback mechanism.

### A rapid activation of defence signalling

The association of a GO term enrichment analysis with a clustering approach enabled us to distinguish different major components of the response. Numerous clusters are enriched with genes related to plant responses to stimuli, either biotic or abiotic. A majority of these genes were differentially expressed at early time-points, showing the ability of trees to react rapidly to mechanical stimuli by modifying the expression of genes involved in the general response to abiotic stresses. These genes encompassed for example an ortholog of the PIA1 PP2C protein phosphatase known to regulate defence responses both positively and negatively in *Arabidopsis* [38], and an ortholog of the *Arabidopsis* CAMTA3 calmodulin-binding TF, which was recently shown to regulate amplitude of Rapid Stress Response Element (RSRE) peak activity in the general stress response, “the initial rapid cascade preceding the induction of consequent stress-specific signalling activities” [39].

In plants, abiotic stresses usually trigger the accumulation of ROS and increase the expression of scavenging enzyme-encoding genes [40]. Here, early responses to bending also included the regulation of genes related to ROS scavenging, such as several peroxidase-encoding genes, or *PtaGrx3* (Potri.017G017300), a member of the glutaredoxin family that regulates protein redox state. It is known that plants can respond to stresses by a coordinated modulation of chloroplast and nuclear gene expression. Such rapid acclimation of plant cell metabolism and photosynthetic machinery is considered a key factor for plant survival. Here, the expression of several genes involved in chloroplast function or development was down-regulated for several hours after the bending. To our knowledge, these results are the first evidence of coordinated modulation following a non-wounding mechanical stimulus.

Taken together, these data suggest that in trees, a transient non-wounding mechanical treatment also quickly activates defence-signalling pathways.

Previous studies have argued that Ca^2+^ may be involved in plant responses to mechanical stimuli [16]. Accordingly, several genes encoding proteins related to calcium such as calmodulins, Calcineurin B-Like calcium sensor proteins (CBLs) and Autoinhibited Ca^2+^-ATPases (ACAs) were also deregulated in poplar stem tissues after bending. The long-term maintenance of the induced expression of *PtaACA13* suggests its role in maintaining Ca^2+^ homeostasis thereby preventing cell injuries or maintaining Ca^2+^ second messenger functions.

Plant hormones are known to play major roles in development and defence against biotic and abiotic stresses, so it was unsurprising to find concomitant modifications in expression of genes related to defence and to hormones at early time-points. In *Arabidopsis*, ethylene and JA are known to strongly contribute to the mechanical signalling pathway in the first-stage responses. Many of the genes studied here encode TF related to ethylene signalling and many others encode proteins involved in JA biosynthesis (Allene Oxide Cyclases, Allene Oxide Synthases, JA-deconjugating enzymes, JA sulfotransferases) or JA signalling (JAZs and “JA-regulated” genes). These observations suggest JA and ethylene are central to mechanoresponse in the poplar model tree, just as they are in ruderal herbs such as *Arabidopsis thaliana*.

### Stem bending elicits transcriptional responses linked to cell wall modification and/or wood development

Poplar stem bending caused a complete cessation of cambial growth during the first hours [4]. The coincidence of early growth cessation in bent poplar and transitory overexpression of genes related to plant defence is consistent with the reported antagonism between plant immunity and growth [41]. In this tradeoff, hormones, especially JA, play a significant role. After the early phase of growth cessation, cambial growth increases during 3 to 5 days before resuming a normal rate of growth. In our study, 10 probesets revealed an increased expression of ribosomal protein-encoding genes at the 24 h PB time-point, which was further confirmed by the clustering approach with cluster 20 showing an enrichment of GO terms related to ribosomes. Sustained accelerated growth, as observed in bent poplar, is heavily reliant on an efficient supply of proteins by the cell machinery [42]. Thus, the effect of bending on the expression of genes encoding ribosomal protein could accompany the strong metabolic activity necessary for accelerated cambial growth.

Although early responses of poplar to a bending stimulus seem to apply to defence and growth, this transcriptomic study indicated that later responses strongly affect cell wall and/or wood development. Indeed, a significant portion of cell wall-related genes displayed differential expression at different time-points. FLA genes were up-regulated at 2 h and 72 h PB while some NAC and MYB encoding genes, known to be part of the transcriptional network that regulates secondary cell formation and wood cell differentiation [43, 44], were first down-regulated at 0.5 h then up-regulated at 72 h PB. Moreover, the most highly induced gene corresponded to a homolog of *Arabidopsis* XTR6, a XTH. In the cell wall, these proteins are thought to specifically catalyse the endolytic cleavage and re-ligation of xyloglucan chains, thus allowing adjustments of cell wall mechanical properties. These observations may indicate the existence of a two-phase process: (i) a relatively rapid response that might be interpreted as a way for plants to modulate growth through adjustment of the expansibility of the primary cell wall followed by (ii) a later response that may set up tissue differentiation and changes in properties to enable acclimation to the encountered mechanical stress. Indeed, multiple stem bendings in poplar were previously shown to modify the mechanical properties (cell wall fraction, elastic stiffness, flexural strength) of the stems [3, 5]. Until now, PtaZFP2 was the only protein putatively involved in such modifications [23]. Our study thus represents a first tentative molecular explanation for this two-phase effect of bending loads.

### A single mechanical stimulus is sufficient to attenuate plant responsiveness to recurrent stimulus

In natural conditions, wind triggers successive bendings of aerial axes. In a previous study, we demonstrated that plants reduce their responsiveness to mechanical signals as a function of their mechanical history —a phenomenon named accommodation [6, 21]. Here, we aimed to unravel the impact of repeated stem bending at genome-wide level by comparing the magnitude of transcriptional responses between two successive bendings and a single bending of the stem. This kinetics is intended to mimic the alternation between windy and quiet days observed in temperate climates [45] more than the frequencies (between 1 and 5 Hz) at which plants oscillate in wind [46]. Our transcriptomic results highlighted the existence of three classes of transcriptional response patterns.

First, the huge majority of these early mechanoresponsive genes (96%) presented a smaller amplitude of regulation 0.5 h after the second bending. Furthermore, the data showed that most of these early-regulated genes returned to their basal expression levels at 24 h PB, at the time when the second bending was applied. The attenuation of transcriptional responses to the second bending thus probably results from a reduction in transcriptional responsiveness and not from saturation of the transcriptional machinery. These data suggest a global desensitization of the first stages of the mechanotransduction pathway.

The description of genes presenting altered responses to successive stimulations has been made elsewhere for other stresses. For example, Ding et al. used RNA-seq analysis to classify drought-responsive genes as “memory genes” or “non-memory” genes as a function of their transcriptional responses to two successive dehydration treatments [47]. Plants can record repeated stimulus and respond in consequence, a phenomena named “attenuation”, “stress imprint” or, as here, “accommodation”. However, contrary to mechanical stem loading, responses are usually enhanced after a second exposure in most of the studies [48]. Whereas gene priming and transcriptional memory are classically thought to help provide an increased, or at least improved, response to a biotic or abiotic stress, accommodation mostly switches off or attenuates the response. Such fine-tuning of the mechanotransduction pathway could help prevent unnecessary thigmomorphogenetic growth reductions while still allowing the plant to become more resistant (acclimated) to the stimulus.

Two unexpected categories of transcriptional responses were observed in our study: (i) the “newly-regulated” genes (19 genes), whose expression was only regulated after the second stimulation, and (ii) the “non-accommodated” genes (44 genes), *i.e.* mechanoresponsive genes that respond similarly to the first and the second stimulus. These data raise the question of the nature of a mechanism, or set of mechanisms, involved in this phenomenon without affecting all the mechanoresponsive genes similarly. As stated in [20], several hypotheses can be put forward: alteration of perception through direct modification of mechanosensors, or a negative feedback control on the mechanotransduction pathway (e.g. through a negative transcription control or epigenetic modifications). The existence of three types of expression profiles makes the hypothesis of a direct control on mechanosensors difficult to consider, except if several types of mechanosensors are involved. Interestingly, a recent study suggested a putative role for FERONIA in mechanosensing [33]. However, it also revealed that the pattern of cytoplasmic changes in [Ca^2+^] observed after a mechanical stimulation cannot be explained solely by FERONIA, which requires other mechanosensors to be involved. In our case, 10% of the “non-accommodated” genes encode proteins possessing a kinase domain, among which the poplar orthologous gene of FERONIA. The non-accommodation of these genes could be a way for the cells to help record recurrent stimuli or to fine-tune cell sensitivity to mechanical stimulation. However, the hypothesis of an active negative feedback control at the transcriptional level also needs to be explored, as suggested by the active inhibition of expression observed at later time-points for most of the early-induced genes.

## Conclusion

This genome-wide study of poplar transcriptional responsiveness to bending brings a novel temporal dimension to our knowledge of plant responses to mechanical stimuli. Moreover, it highlights the extent of the accommodation process and the existence of diverse gene regulation types in response to recurring mechanically stressing events. The identification of the underlying molecular mechanisms opens news perspectives for gaining deeper understanding of how the intensity and recurrence of a stimulus affect plant growth and development. We anticipate this study as a start point to the generation of predictive models of the gene regulatory networks mediating poplar transcriptional responses to mechanical stimulation.

## Methods

### Plant material, culture conditions and bending treatments

Wild-type poplars (*Populus tremula* × *P. alba* cv 717-1B4) were obtained by *in vitro* micropropagation on MS medium [49]. After acclimation, plants were grown in liquid nutrient solution [50] in a growth chamber at 22 °C, with a relative air humidity of 60% and a 16 h/8 h light/dark cycle under photosynthetic active radiation (PAR) of 50 μmol.m^−2^.s^−1^.

Analyses were conducted on 3-month-old poplars. Bendings were realized according to [23]. Briefly, each basal stem part was bent transiently (10 s) against a plastic tube so as to apply controlled and quantified flexural strains as described in [4] (Additional file 6).

Two different types of bending treatment were applied. The first was a single bending treatment, for which the bent portion of the stem was collected from bent and control plants at 0.5, 2, 24 and 72 h after the mechanical stimulation, thus yielding a time-series expression dataset.

The second type of treatment consisted of two successive transient bendings separated by a 24 h time interval, for which the bent portion of the stem was collected at 0.5 h after the second mechanical stimulation.

### Microarray analysis

Three independent experiments (*i.e.* three biological replicates) on two individuals per experiment and per condition were analysed using Affymetrix GeneChip® Poplar Genome Array oligonucleotide microarrays (Santa Clare, Canada).

RNA was isolated from 100 mg of bent or unbent stem using the RNeasy Plant Mini kit followed by a DNAse1 treatment according to the manufacturer’s instruction (Qiagen,Germany). RNA sample integrity was checked on an Agilent 2100 Bioanalyzer (Agilent Technologies, Germany). The amplification, labelling, hybridization and imaging procedures were performed according to the manufacturer’s instructions [51] at the URGV Transcriptomics Platform (Evry, France).

For each independent experiment, arrays were hybridized with complementary RNA obtained from 2 μg of total RNA made of a pool from two individuals. Arrays were scanned with the GeneChip 3000-7G Scanner driven by the GeneChip Operating Software (GCOS). Raw data were imported into R software for analysis and normalized by the GC-RMA algorithm [52] available in the Bioconductor package.

A two-group t-test assuming equal variance between groups was performed to determine which genes were differentially expressed in the following comparisons: (i) unbent *vs.* bent stems at each time-point of the time-series, (ii) stems bent once and harvested 0.5 h after the mechanical stimulation *vs.* stems bent twice, (iii) stems bent once and harvested 24 h after the mechanical stimulation *vs.* stems bent twice. To fit the assumption of equal variance of gene expression between groups, genes displaying extreme variances (too small or too large) were excluded. The raw *P*-values were adjusted by the Benjamini–Hochberg procedure [53], which controls false discovery rate. A probeset was declared as representing differential expression of a gene if Benjamini–Hochberg *P*-value was < 0.05. All the raw and normalized data are available through the Gene Expression Omnibus repository at the NCBI [GEO submission GSE44321; [54]]. In order to limit the tendency of this Benjamini–Hochberg correction to lead to false negatives, the data was pre-treated prior to statistical analysis. When a probeset presented a log-transformed intensity under the threshold of 4 for each repeated sample in one comparison, it was considered as unexpressed and these samples were removed from the analysis.

### Microarray probeset selection and annotation up-dating

The Affymetrix 61 k poplar microarray (composed of 61,251 probesets) was designed on the first version of *Populus Trichocarpa* genome, but also on mRNA and EST from different species of *Populus* (e.g. *P. tremula*, *P. deltoids*, *P. alba*) [51]. Consequently, some genes are targeted by more than one probeset. In the Affymetrix poplar GeneChip, only 585 probesets corresponded to sequences of the *P. alba* x *P. tremula* hybrid used in our analysis. To reduce multiple gene probe elements without impacting the global analysis, probesets designed from *P. alba*, *P. tremula* and *P. trichocarpa* (the sequenced genome) and hybrids from these 3 species were also included in this analysis —representing 57,654 probesets of the 61,251 initial probesets of the Affymetrix 61 k poplar microarray.

To update the Affymetrix probeset annotations to the third version of the Phytozome *P. trichocarpa* genome, a BLASTn search was performed between the probeset sequences and the third version of *P. trichocarpa* sequencing (ncbi-blast-2.2.28 – 2013.04.01 version). 50,691 of the selected probesets were successfully associated to 31,121 different gene IDs among the 41,335 genes of the third version of *P. trichocarpa* genome. The *Arabidopsis thaliana* ortholog of each *P. trichocarpa* gene targeted by a probeset was retrieved in the Phytozome database of the third version of the *P. trichocarpa* genome (file: Ptrichocarpa_210_annotation_info.txt) as “best *Arabidopsis* TAIR10 hit symbol” [55].

### Clustering of the microarray data

To identify groups of genes presenting similar patterns of expression in response to one bending and/or similar expression behaviour toward a second bending, we performed k-means clustering on the DEG (*i.e.* genes that are differentially expressed in at least one condition). We used the Euclidian metric to calculate the distances between the DEG, as follows. Let $x$ and $y$ two genes defined by $n$ values (and $i$ one of these $n$ values). The distance between $x$ and $y$ is given by $d\left( x, y\right)=\sqrt{\sum_{i=1}^n\left({x}_i-{y}_i\right)2}$. Three types of $n$ values were chosen. Two of them allowed genes to group according to their pattern of expression along the time-course: (i) the speed of transcript accumulation between the different time points, given by $v\left({x}_{\varDelta t}\right)=\frac{a_{t+1}+{a}_t}{{\varDelta t}_{\left( t, t+1\right)}}$ where ${a}_{t+1}$ and ${a}_t$ are the differences of expression measured for gene $x$ at $t+1$ and $t$, and $\varDelta t$ the lapse of time between the two time-points. The 0 h PB and 120 h PB time-points were also used and their level of differential expression was set to 0; (ii) a scale grade allowing discrimination between significant and non-significant differential expression values and expressing the type of regulation: 1 for a significant up-regulation, −1 for a significant down-regulation, and 0 for a non-significant regulation. The third type of n value, corresponding to the subtraction of expression ratios observed after one bending to expression ratios observed after two bendings, aimed to group genes according to their behaviour in response to the second bending.

### GO enrichment analysis

To categorize the DEG based on their biological functions, GO enrichment was searched for each expression cluster. In the third version of the poplar genome, genes are associated to a narrow numbers of annotations – e.g. *PtaZFP2* (Potri.001G235800) is annotated with 2 GO terms in the Phytozome database whereas its homologous *A. thaliana* gene has 10 GO annotations on Phytozome. Consequently, the GO enrichment of each cluster was performed with the term-enrichment tool from Amigo (1.8) using the *A. thaliana* orthologs and the TAIR database as a background. The maximum-thresholds *P*-value was set at 0.05 but most of the clusters presented *P*-values under 0.01.

### Selection of genes as representatives of each cluster

For each cluster, one gene representing the other genes of the cluster was selected based on two criteria: (i) its expression should be as close as possible to the median expression value of a cluster; (ii) it should be representative of either the GO functional category enrichment found for the cluster or a well-known component of the mechanical signalling pathway.

### Real-time quantitative RT-PCR experiments on candidate genes

The time-course analysis of candidate genes expression was performed via two independent experiments (*i.e.* two biological replicates) where young poplars were treated following the conditions described for microarray analysis. For each time-point, each independent experiment includes two mechanical-stimulated plants and one control. The bent portion of the poplar stem was collected 1, 2, 5, 9, 13, 17, and 21 h after mechanical stimulation.

Total RNAs was extracted from about 100 mg of bent stems using CTAB extraction buffer as described in Chang et al. [56] then treated with RNase-free RQ1 DNase (Promega, Charbonnières-les-Bains, France). RNA was quantified spectrophotometrically using a NanoDrop apparatus (Thermo Fisher Scientific Inc., Waltham, MA) and checked by agarose gel electrophoresis. First-strand cDNA was synthesized from 1 μg total RNAs using oligodT and SuperScript III (Invitrogen, Cergy-Pontoise, France). The real-time quantitative RT–PCR amplifications were performed using an iCycler IQ (Bio-Rad) with SYBR green as fluorescent dye. Each PCR reaction (15 μL) contained the following: cDNA (4 μL of a 1:40 dilution of the first cDNA strands), MESA GREEN qPCR MasterMix Plus for SYBR® Assay w/ fluorescein (Eurogentec, Angers, France) (1×) and primers (200nM of each). After a heat step at 95 °C for 3 min, PCR cycling conditions were 40 cycles of denaturation (95 °C, 10 s), annealing (temperature according to primers, Additional file 4, 10 s) and elongation (72 °C, 15 s), ending with a melt curve (increment of 0.5 °C each cycle from primer tm to 95 °C). Transcripts of each studied gene and reference genes were amplified using the primers described in Additional file 4. Specificity of amplification was confirmed by (i) determining the melt curves for the PCR products, (ii) gel electrophoresis and (iii) sequencing. An Index corresponding to the expression of 5 reference genes (*EF-1α*, *UP1*, *UP2*, *TIP41* and *UBC*) was calculated. The Quantitative Relative Normalized (QRN) abundance of each gene transcript was calculated by comparison between bent and control plants and by normalizing with the expression of the index using the delta–delta method mathematical model [57].

Each run of the real-time PCR amplifications was carried out in triplicate. RNAs samples used for microarray, arising from control plants or plants collected 0.5, 2, or 24 h after bending, were also included in this Q-PCR analysis.

## Additional files

Additional file 1: Table S1. List of genes differentially expressed probset in response to one or two bendings, as identified by microarray analysis. Affymetrix 61 k poplar microarrays (composed of 61,251 probesets) were used to detect differences in expression. Four comparisons between bent plants (b) *vs.* control plants (c) at different time points after the bending were realized: 0.5 h PB (1b0.5 h *vs.* C0.5 h), 2 h PB (1b2h *vs.* C2h), 24 h PB (1b24h *vs.* C24h) and 72 h PB (1b72h *vs.* C72h). To understand the effect of a second bending, we ran two comparisons (i) 0.5 h after the 2nd bending (with a time-lapse of 24 h between the two bendings) compared to 24 h after the 1st bending (2b0.5 h *vs.* 1b24h) and (ii) 0.5 h after the 2nd bending compared to 0.5 h after the 1st bending (2b0.5 h *vs.* 1b0.5 h). Expression was considered significantly different if the *P*-value from the Benjamini–Hochberg test (BH *P*-value) is below 0.05. (CSV 694 kb)

Additional file 2: Table S2. List of the 10 most up- or down-regulated genes at each time-point. *gene_ID corresponds to the gene model found in Phytozome database [55] for poplar or *Arabidopsis* (Ath). The log2 expression fold-change is shown for the different time-points: 0.5 h (exp 0.5 h), 2 h (exp 2 h), 24 h (exp 24 h) and 72 h (exp 72 h). (XLSX 15 kb)

Additional file 3: Table S3. Gene Ontology enrichment analysis applied to the clusters. The GO enrichment was performed using the Amigo (1.8) term enrichment tool [58]. The list of *A. thaliana* orthologs of each cluster was used, and the TAIR database was chosen as a background. The maximum *P*-value for the threshold was 0.01, except for clusters marked * and ** for which maximum *P*-value was set at 0.05 and 0.1, respectively. (XLSX 13 kb)

Additional file 4: Table S4. Description of the genes analysed in the refined time-course analysis. Primer sequences are described. *Id_gene corresponds to the gene model found in the Phytozome database [55] for poplar or *Arabidopsis* (Ath). **Poplar gene name was attributed arbitrarily following the gene function described for the *Arabidopsis* homologous gene in Phytozome. Gene membership to expression clusters is specified. (XLSX 14 kb)

Additional file 5: Table S5. List of “newly regulated” genes after two successive bendings. These genes are not regulated 0.5 h after a single stem bending, but they do become regulated 0.5 h after a second bending (occurring 24 h after the 1st one). *Id_gene corresponds to the gene model found in the Phytozome database [55] for poplar or *Arabidopsis* (Ath). Log2 expression fold-change is shown: expression fold-change 0.5 h after a single bending compared to a control plant (1B0.5 h/Control) and expression fold-change 0.5 h after a second bending compared to the 24 h PB time-point (2B0.5 h/1B24h). (XLSX 11 kb)

Additional file 6: Figure S1. Bending device. The stem is transitory –10s back and forth– pushed against a plastic tube of known diameter; the stem is thus locally bent around the tube. Therefore, the stem is subjected to a quantified curvature in terms of strains. Locally, the applied strain is the product of the curvature of the central line and the stem radius (r_stem_). In the case of small curvature, the curvature of the central line is given by the inverse of the sum of the stem radius and of the radius of the plastic tube (r_tube_). (Modified from [4]). (PDF 747 kb)

## Abbreviations

BP: Biological process
DEG: Differentially expressed genes
ERF: Ethylene response factor
GO: Gene Ontology
JA: Jasmonic acid
MF: Molecular function
MS: MechanoSensitive
PB: Post-bending
RLK: Receptor-like kinase
ROS: Reactive oxygen species
TF: Transcription factor
XTH: Xyloglucan endo-Transglycosylase/Hydrolase
