# Supplementary figures and images for: Poplar stem transcriptome is massively remodelled in response to single or repeated mechanical stimuli
Source: BMC Genomics. 2017 Apr 17;18:300. doi: 10.1186/s12864-017-3670-1 (PMC5392906; doi:10.1186/s12864-017-3670-1)

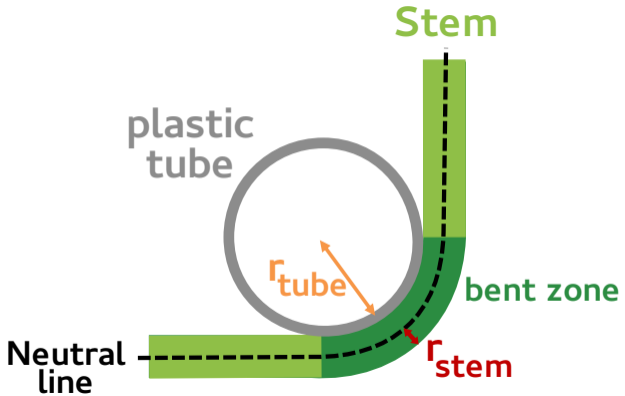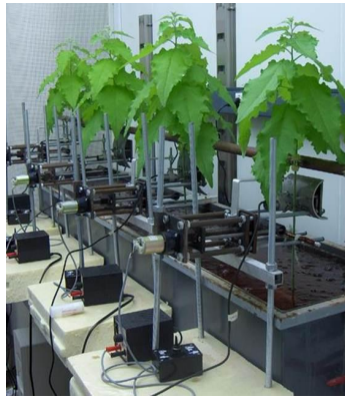

Supplement: Supplementary file 6 — Bending device. The stem is transitory –10s back and forth– pushed against a plastic tube of known diameter; the stem is thus locally bent around the tube. Therefore, the stem is subjected to a quantified curvature in terms of strains. Locally, the applied strain is the product of the curvature of the central line and the stem radius (rstem). In the case of small curvature, the curvature of the central line is given by the inverse of the sum of the stem radius and of the radius of the plastic tube (rtube). (Modified from [4]). (PDF 747 kb) [file 12864_2017_3670_MOESM6_ESM.pdf]
